# Cyr61 promotes Schwann cell proliferation and migration via αvβ3 integrin

**DOI:** 10.1186/s12860-021-00360-y

**Published:** 2021-04-07

**Authors:** Zhenghui Cheng, Yawen Zhang, Yinchao Tian, Yuhan Chen, Fei Ding, Han Wu, Yuhua Ji, Mi Shen

**Affiliations:** 1 Key Laboratory of Neuroregeneration of Jiangsu and Ministry of Education, Co-innovation Center of Neuroregeneration, NMPA Key Laboratory for Research and Evaluation of Tissue Engineering Technology Products, Nantong University, Nantong, 226001 People’s Republic of China; 2 Jiangsu Clinical Medicine Center of Tissue Engineering and Nerve Injury Repair, Nantong, 226001 People’s Republic of China; 3 Department of General Surgery, Affiliated Hospital of Nantong University, Nantong, Jiangsu 226001 People’s Republic of China

**Keywords:** Schwann cells, Cyr61, Proliferation, Migration

## Abstract

**Background:**

Schwann cells (SCs) play a crucial role in the repair of peripheral nerves. This is due to their ability to proliferate, migrate, and provide trophic support to axon regrowth. During peripheral nerve injury, SCs de-differentiate and reprogram to gain the ability to repair nerves. Cysteine-rich 61 (Cyr61/CCN1) is a member of the CCN family of matrix cell proteins and have been reported to be abundant in the secretome of repair mediating SCs. In this study we investigate the function of Cyr61 in SCs.

**Results:**

We observed Cyr61 was expressed both in vivo and in vitro*.* The promoting effect of Cyr61 on SC proliferation and migration was through autocrine and paracrine mechanisms. SCs expressed αvβ3 integrin and the effect of Cyr61 on SC proliferation and migration could be blocked via αvβ3 integrin. Cyr61 could influence c-Jun protein expression in cultured SCs.

**Conclusions:**

In this study, we found that Cyr61 promotes SC proliferation and migration via αvβ3 integrin and regulates c-Jun expression. Our study contributes to the understanding of cellular and molecular mechanisms underlying SC’s function during nerve injury, and thus, may facilitate the regeneration of peripheral nerves after injury.

**Supplementary Information:**

The online version contains supplementary material available at 10.1186/s12860-021-00360-y.

## Background

Peripheral nerve injury is a common clinical problem. It seriously affects the quality of life in patients and results in social and economic burdens. The treatment for peripheral nerve injury includes nerve suturing, autogenous nerve transplantation, and tissue-engineered nerve transplantation. These treatments promote the functional recovery of injured nerves [1]. However, to date, the clinical effects of these therapies have not been satisfactory. Understanding the cellular and molecular mechanisms of peripheral nerve injury will be helpful for the clinical treatment of peripheral nerve injury.

Compared to peripheral nerve injury in the central nervous system (CNS), SCs are the main glial cells in peripheral nerves and have a robust ability to regenerate [2]. Following peripheral nerve injury, SCs start to proliferate and migrate to the injured site to clear axon and myelin debris and build bands of Büngner [3]. In addition, SCs secrete a large number of neurotrophic factors to support the survival of neurons and create a conducive microenvironment for nerve regeneration [4]. These events rely on the remarkable ability of SCs to transform into a potent repair phenotype. SCs de-differentiate into a proliferative, immature-like state via the activation of the JUN dependent repair program [5]. After peripheral nerve injury, axon breaks, and SCs lose contact with their axons. SCs can survive in the absence of axons which is important for subsequent nerve regeneration. This is in part because of the ability of SCs to support their survival through autocrine mechanisms [6]. Factors that accelerate SCs proliferation during the early stages after peripheral nerve injury and/or promote SCs migration and myelination during the later stages after nerve injury benefit nerve regeneration and functional recovery [3]. However, the precise mechanisms for this are unclear. Considering the critical role played by SCs, identifying factors that can accelerate the proliferation and migration of SCs may help promote the repair and regeneration of peripheral nerves after injury.

Cysteine-rich protein 61 (Cyr61, also known as CCN1) is a member of the CCN family of matrix cell proteins. Cyr61 is a secretory protein of the CCN family signal protein related to ECM [7]. It can regulate a wide range of cell activities, including cell adhesion, migration, proliferation, differentiation, apoptosis, and aging by interacting with integrin receptors on the cell surface [8]. Previous studies have demonstrated that Cyr61 stimulates the migration of smooth muscle cells [9], fibroblasts [10], endothelial cells [11], and some cancer cells [12–14]. Cyr61 has also been observed in the nervous system. For example, Cyr61, as a dendrite growth regulator of hippocampal neurons, controls dendrite growth in an αβ_1_ integrin-dependent manner [15]. Cyr61 also plays a role in tissue repair. During the process of skin wound healing, Cyr61 can accelerate re-epithelialization by promoting the migration and proliferation of keratinocytes [16]. A recent study found that SCs transformed into repair mediating SCs after FYT702P treatment and the secretion levels of Cyr61 in SC conditioned medium increased. This indicated that Cyr61 may participate in SCs biology to facilitate nerve repair [17]. However, its role in SCs has not been fully elucidated.

In this study, we aimed to demonstrate the functional effects of Cyr61 on SCs proliferation and migration. We found that Cyr61 affects proliferation and migration in SCs through autocrine and paracrine mechanisms, and functions via αvβ3 integrin expressed on SCs and regulating c-Jun expression. Together, these findings suggest that Cyr61 may contribute to peripheral nerve system (PNS) repair by supporting SC proliferation and migration important for nerve regeneration.

## Results

### In vivo and in vitro Cyr61 expression in SCs

**Fig. 1:**
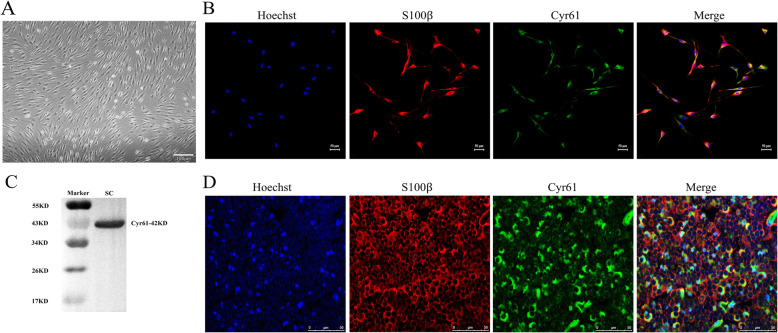
In vivo and vitro expression of Cyr61. **a** Primary SC cultures under light microscopy; **b** SC primary cultures were immunostained using antibodies against Cyr61 (green color) and S100β (red color), with cell nuclei stained using Hoechst 33342 (blue color), Scale bar = 50 μm; **c** Western blots demonstrating Cyr61 protein expression in primary cultured SCs; **d** nerve transverse sections immunostained using antibodies against Cyr61 (green color) and S100β (red color), with cell nuclei stained using Hoechst 33342 (blue color), Scale bar = 50 μm

Western blot and immunocytofluorescence (ICF) assays were used to determine the expression levels of Cyr61 in cultured SCs. Primary cultured SCs are shown in Fig. 1a. Under light microscopy, the cells were bright and arranged in a regular pattern and depicted a typical SC morphology. In cultured SCs, ICF assays using anti-Cyr61 and the SC marker anti-S100β antibodies demonstrated that Cyr61 expression overlapped with S100β (Fig. 1b). Western blot also demonstrated that Cyr61 protein (42KD) was expressed in cultured SCs (Fig. 1c). In transverse sections of normal rat sciatic nerves, immunohistofluorescence (IHF) assays using anti-Cyr61 and anti-S100β antibodies demonstrated that Cyr61 protein was colocalized with S100β protein expression (Fig. 1d).

### Inhibition of endogenous Cyr61 expression levels in SCs attenuates cell proliferation and migration and downregulate c-Jun expression in SCs

**Fig. 2:**
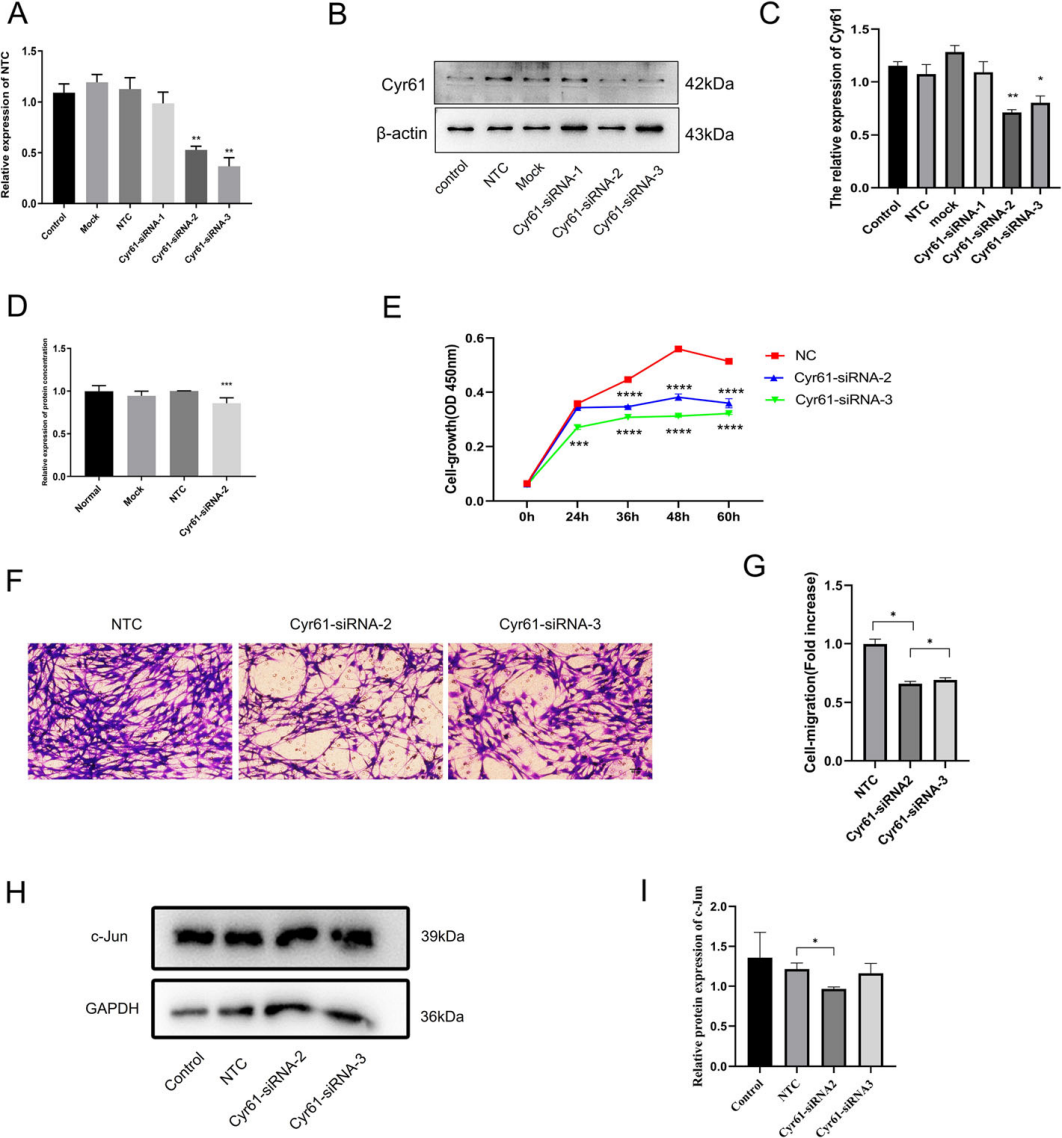
Endogenous Cyr61 is required for the proliferation and migration of SCs and regulated c-Jun expression. **a** Histograms for three independent qPCR experiments of primary SCs transfected with Cyr61-specific siRNAs or with NTC siRNA; **b** Western blots demonstrating protein knockdown efficiency of three different Cyr61-targeting siRNAs; **c** Histograms for three independent western blot experiments of primary SCs were transfected with Cyr61-specific siRNAs or with NTC siRNA; **d** ELISA histograms of the results from three independent experiments for Cyr61 secreted by SCs after transfection with Cyr61-specific siRNA or with NTC siRNA; **e** Histograms of three independent experiments of SCs cultured for 60 h after transfection with Cyr61-specific siRNAs or with NTC siRNA; **f** Representative images of SCs transfected with siRNAs that migrated to the underside of transwell membranes. **g** Histogram results from three independent experiments for cell migration. **h** Western blots demonstrating c-Jun protein expression in SCs and SCs transfected NTC siRNA or Cyr61-specific siRNAs. **i** Histograms for three independent western blot experiments of c-Jun expression in SCs and SCs transfected with Cyr61-specific siRNAs or with NTC siRNA. *, *p* < 0.05, **, *p* < 0.01, ***, *p* < 0.001, ****, *p* < 0.0001

SCs were transfected with siRNA against Cyr61 to determine whether reduced Cyr61 expression levels affect cell proliferation and migration. Three siRNAs designated as Cyr61-siRNA-1, Cyr61-siRNA-2, Cyr61-siRNA-3 were designed to reduce expression levels of Cyr61. The qPCR demonstrated that no changes in Cyr61 mRNA levels were observed in cells transfected with non-targeting negative control (NTC) siRNA compared to mock-transfected cells (Fig. 2a). The western blot demonstrated that no changes in Cyr61 protein levels were observed in cells transfected with non-targeting negative control (NTC) siRNA compared to mock-transfected and control cells (Fig. 2b). Compared to cells transfected with NTC siRNA, SCs transfected with siRNA-2 or siRNA-3 had reduced Cyr61 mRNA and protein expression levels (Fig. 2a-c). The secretion levels of Cyr61 have no changes in SCs transfected with NTC siRNA compared to mock-transfected SCs and the secretion levels of Cyr61 in SCs after transfection with Cyr61-siRNA2 were lower compared to SCs transfected with NTC, as determined by ELISA (Fig. 2d). This indicated that Cyr61-siRNA transfection inhibited Cyr61 secretion in SCs. Cell Counting Kit8 (CCK-8) assays demonstrated that proliferation rates of SCs transfected with Cyr61 siRNA-2 or Cyr61 siRNA-3 were also lower compared to cells transfected with NTC siRNA at different time points from 0 to 60 h (Fig. 2e). Transwell-based migration assays and crystal violet staining were then used to determine the effects of Cyr61-siRNA-2 or Cyr61-siRNA-3 on SC migration. The number of cells that migrated through the transwell chamber, assessed using crystal violet staining, was substantially lower in SCs transfected with Cyr61-siRNA-2 or Cyr61-siRNA-3 compared to SCs transfected with NTC siRNA (Fig. 2f and g). This indicated that Cyr61 siRNA transfection suppressed cell migration. C-Jun is essential for the normal activation of the SC repair programme and contributes to SC proliferation and migration [18–20]. Thun, western blot was used to detect c-Jun expression. The results showed demonstrated that compared to SCs transfected with NTC siRNA c-Jun protein expression was lower in SCs transfected with Cyr61- siRNA-2 while not significant in SCs transfected with Cyr61-siRNA-3 (Fig. 2h and i).

### Cyr61 enhances SCs proliferation and migration and enhanced c-Jun expression in SCs

**Fig. 3:**
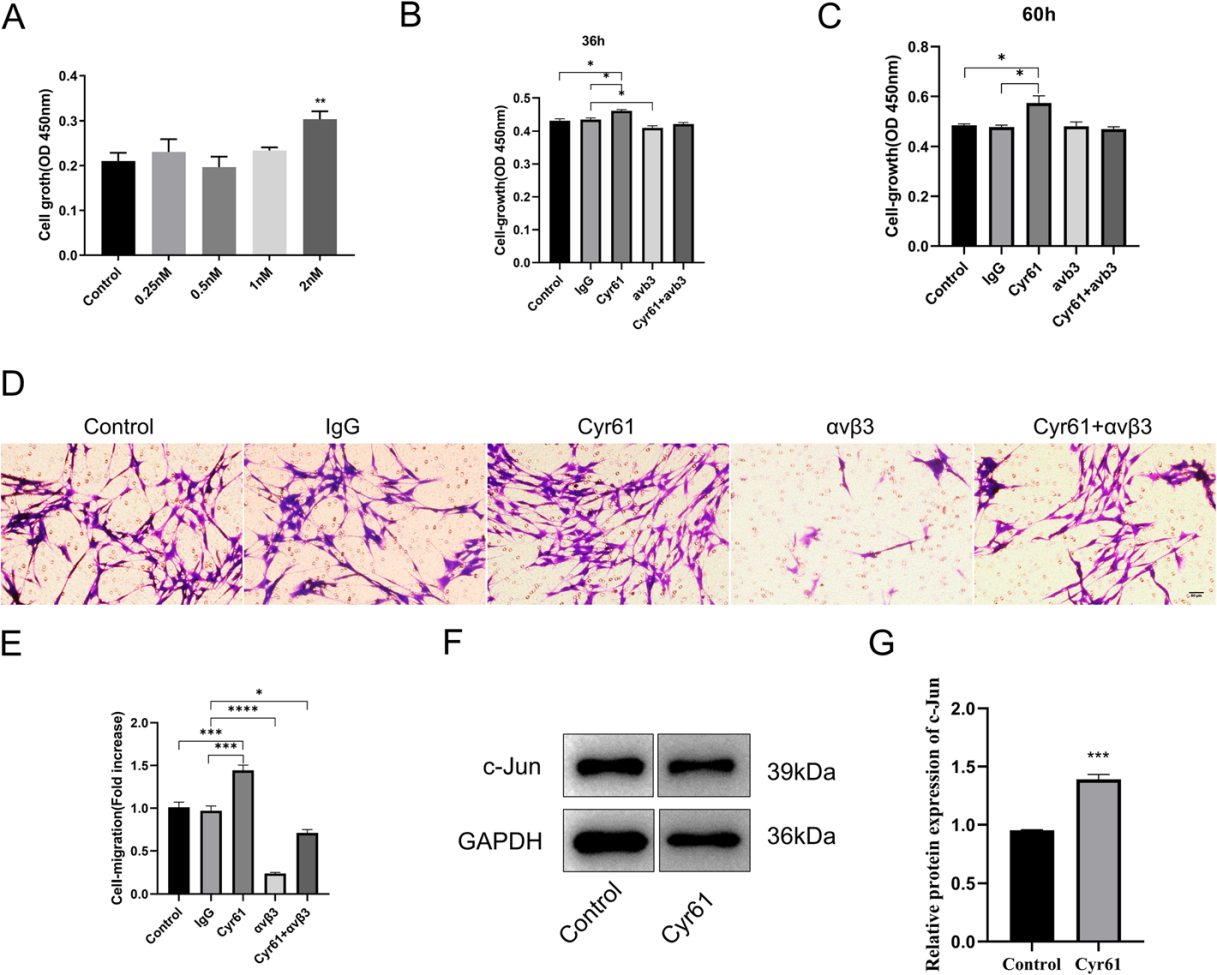
Exogenous Cyr61 increases the proliferation and migration of SCs and upregulated c-Jun expression. **a** Histogram of SC proliferation cultured for 48 h after the addition of 0.25 nM, 0.5 nM, 1 nM, 2 nM of Cyr61 and control media measured using CCK-8 assays; **b** Histogram of SC proliferation cultured for 36 h after the addition of 2 nM of Cyr61, 0.5 μg neutralizing antibody αvβ3 and Mouse IgG1 Isotype Control (IgG), 2 nM Cyr61 + 0.5μg neutralizing antibody αvβ3 and control media measured using CCK-8 assays; **c** Histogram of SC proliferation cultured for 60 h after the addition of 2 nM of Cyr61, 0.5 μg neutralizing antibody αvβ3 and Mouse IgG1 Isotype Control (IgG), 2 nM Cyr61 + 0.5 μg neutralizing antibody αvβ3 and control media measured using CCK-8 assays; **d** Representative images of SCs treated with 2 nM of Cyr61, 0.5 μg neutralizing antibody αvβ3 and Mouse IgG1 Isotype Control (IgG), 2 nM Cyr61 + 0.5 μg neutralizing antibody αvβ3 and control media that migrated to the underside of the transwell membrane after 24 h. **e** Histograms from three independent cell migration experiments; **f** Western blots demonstrating c-Jun protein expression in SCs treated with 2 nM of Cyr61 or control medium; **i** Histograms for three independent western blot experiments of c-Jun expression in SCs treated with 2 nM of Cyr61 or control medium. *, *p* < 0.05, **, *p* < 0.01, ***, *p* < 0.001, ****, *p* < 0.0001

Cyr61 has been reported to stimulate proliferation and migration of cancer cells, fibroblasts, and endothelial cells [21], however, the effect of Cyr61 in SCs remained to be deciphered. To determine whether Cyr61 influences the proliferation and migration of SCs, CCK-8 assays, and transwell-based migration assays were performed. To determine the effective concentration of Cyr61 on SCs, different concentrations of Cyr61 on SC proliferation was measured using CCK-8 assays. As shown in Fig. 3a, when 2 nM exogenous Cyr61 was included in the culture media, the SC proliferation rate increased compared to control cells after 48 h. This result indicated that the proliferation of cultured SCs could be increased by exogenous Cyr61. Transwell assays were then performed to measure cell migration. Cells that were able to migrate to the lower chamber were quantitated to determine the effects of exogenous Cyr61. Results of the migration assays demonstrated that 2 nM Cyr61 added to the lower chamber of the transwell increased the migration rate of SCs compared to media alone (Fig. 3d and e). Results of Western blot demonstrated that the protein expression of c-Jun in SCs was increased after adding exogenous Cyr61(Fig. 3f and g). This might suggest that Cyr61 could increase the proliferation and migration of SCs and increased c-Jun protein expression in SCs.

### Cyr61 enhances SC proliferation and migration via integrin αvβ3

**Fig. 4:**
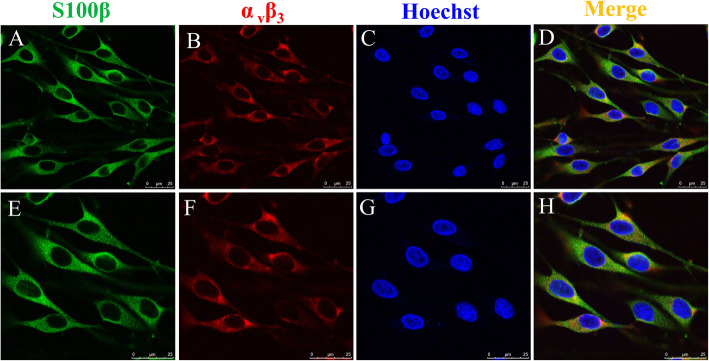
αvβ3 expression levels in SCs determined using ICF. Primary SCs were immunostained using antibodies against S100β (green color) (**a, e**) and αvβ3 (red color) (**b, f**), cell nuclei were stained using Hoechst 33342 (blue color) (**c, f**), **a-d**: Scale bar = 50 μm, e-h: Scale bar = 25 μm

As a secreted protein, Cyr61 binds to membrane receptors on the cell surface. Several studies have shown that αvβ3 integrin is a cell receptor for Cyr61. Cyr61 associates with αvβ3 integrin to promote endothelial cell adhesion, migration, proliferation, survival, and tubular formation [13]. Cyr61 modulates vascular formation by directly binding to αvβ3 to enhance endothelial cell adhesion, migration, and proliferation [19]. In addition, Cyr61 has been shown to direct chondrosarcoma cell migration through αvβ3 integrin [20]. However, the function of Cyr61 on SCs is unknown. Hence, immunofluorescence assays were used to determine the expression of αvβ3 integrin in SCs. The αvβ3 was expressed in S100β-positive cells, which indicated that αvβ3 was expressed in SCs (Fig. 4).

To determine whether Cyr61 influences the proliferation and migration of SCs via αvβ3 integrin, CCK-8, and transwell-based migration assays were performed. As shown in Fig. 3b-e, the proliferation and migration rate of SCs was inhibited by αvβ3 neutralizing antibody, and the increased proliferation and migration rate of SCs with addition of Cyr61 was blocked by αvβ3 neutralizing antibody. The proliferation rate of SCs showed no difference by αvβ3 neutralizing antibody after cultured for 60 h (Fig. 3c). CCK-8 assays demonstrated that 2 nM of Cyr61 could increase the proliferation of SCs, but this effect could be inhibited by 0.5 μg αvβ3 neutralizing antibody after cultured for 36 h and 60 h (Fig. 3b and d). The number of cells that migrated through the transwell chamber was assessed using crystal violet staining (Fig. 3e). The addition of exogenous Cyr61 increased SC migration and this effect could be inhibited by 0.5 μg αvβ3 neutralizing antibody. The addition of αvβ3 neutralizing antibody decreased SC migration rates (Fig. 3e). These results indicated that αvβ3 integrin was involved in SCs proliferation and migration and Cyr61 could increase the proliferation and migration of SCs via αvβ3.

## Discussion

A better understanding of factors that facilitates nerve repair is essential for future improvement in regenerative medicine [5]. Our findings indicate that SCs express Cyr61 both in vivo and in vitro. We demonstrated that inhibition of Cyr61 expression in SCs can attenuate SC proliferation and migration ability. Cyr61 enhances SC proliferation and migration via αvβ3 integrin. Cyr61 enhances c-Jun expression in cultured SCs. Silencing Cyr61 in cultured SCs could decrease c-Jun expression.

The unique regeneration ability of PNS is attributed to the function of SCs. Glial cells in the peripheral nerve have robust plasticity [22]. In addition, SCs secrete a variety of factors to create a microenvironment for cell regeneration, including nerve growth factors [23]. Neuroregulatory proteins secreted by SCs induce an autocrine mechanism to promote SC proliferation [24]. SC survival after an injury is regulated by an autocrine survival loop that includes the secretion of IGF-1, platelet-derived growth factor-BB, and NT-3 [25]. Understanding how secreted proteins from SC influences its function may contribute to the mechanistic understanding of nerve regeneration.

Previous studies have demonstrated increased Cyr61 secretion by repair mediating SCs. The repair type SCs produce guidance tracks for regenerating axons called Büngner bands. Once SCs reprogrammed into repair type cells, precursor/immature SC properties are restored. Its proliferation and migration abilities increase. However, the mechanism is yet to be deciphered. Reprograming SCs into repair phenotype is controlled transcriptionally by mechanisms involving the transcription factor c-Jun, which is rapidly upregulated in SCs after nerve injury [4, 26]. In this study, primary SCs were cultured in vitro to determine the effects of Cyr61 on the proliferation and migration of SCs. Our results demonstrated that compared to cells transfected with NTC siRNA, Cyr61 secretion in cells transfected with cyr61 siRNA was reduced. In addition, the proliferation and migration ability of SCs transfected with Cyr61 siRNA was reduced significantly. Silencing Cyr61 in cultured SCs also downregulated c-Jun expression. This suggested that lower Cyr61 secretion and expression in SCs leads to reduced proliferation and migration rates. And with the decreasing of Cyr61 in cultured SCs, c-Jun expression was inhibited. We next cultured SCs with exogenous Cyr61 to observe its biological effect. Our results indicated that exogenous Cyr61 could increase the proliferation and migration of SCs. In the meantime, c-Jun expression was increased in cultured SCs with exogenous Cyr61. These results suggested that Cyr61, through an autocrine or paracrine mechanism, could significantly increase the proliferation and migration of SCs. Cyr61 could also influence the c-Jun expression in cultured SCs. It has long been known that c-Jun is rapidly induced to high levels in the SCs of injured nerves [4]. C-Jun promotes dedifferentiation of SCs and overexpression of c-Jun alone might be sufficient to reprogram SCs of intact nerves into repair phenotype [27]. C-Jun signaling involved in promoting many cell proliferation and migration [20, 28, 29]. C-Jun-modified SCs showed enhanced proliferation and migration abilities [20]. Here, we suppose that Cyr61 promotes SC proliferation and migration by regulating c-Jun expression. Since increased secretion of Cyr61 was found in repair mediating SCs, Cyr61 might also could modulate SC phenotype by influence c-Jun protein expression. During the process of peripheral nerve regeneration after injury, we hypothesized that SC repair type cells can survive in an environment that lacks the support of axons and other cells. One of the reasons could be that this is through their ability to proliferate and migrate via the secretion of Cyr61.

Cyr61 has been previously demonstrated to regulate cell proliferation and migration by binding to integrin receptors on the cell surface. Exogenous recombinant Cyr61 has been reported to induce angiogenesis [30] and promote cell proliferation, migration, adhesion, and differentiation [31]. At present, the known receptors for Cyr61 include integrin α_6_β_11_ [32, 33], α_IIb_β_3_ [34], α_m_β_2_ [35], αvβ3 [36, 37], β_1_ [38], α_D_β_2_ [39], and heparinase (HSPGs) [9]. Cyr61 can associate with integrin αvβ3 on the surface of endothelial cells to promote endothelial cell adhesion, migration, proliferation, survival, and tubular formation [11]. By associating with integrin α_v_β_5_ and αvβ3 on bile duct cells, Cyr61 can induce the expansion of the bile duct [40]. In addition, Cyr61 can synergize with other mitogenic growth factors to enhance growth factor-induced DNA synthesis in fibroblasts and endothelial cells through integrin αvβ3 [10, 41]. But the function of Cyr61 with integrin αvβ3 on SCs is unclear. Using ICF, we demonstrated that SCs expressed the receptor α_V_β_3_. A recent study showed that SPP1 might promotes SCs proliferation by binding receptors αvβ3 [42]. In our research, the ability of Cyr61 to promote the proliferation and migration of SCs was reduced by blocking the αvβ3 receptor. This strongly suggested that Cyr61 played a significant role in SC proliferation and migration through the integrin receptor αvβ3, and we hypothesize this was via an autocrine mechanism.

## Conclusions

In summary, we demonstrated that Cyr61 promotes proliferation and migration of SCs through an autocrine or paracrine mechanism via αvβ3 integrin. Cyr61 might modulate SC function by regulating c-Jun expression. Our results provide a functional mechanism of SC secreted proteins to promote nerve regeneration which may provide strategies for nerve repair.

## Methods

### Animals

Thirty neonatal 1 to 3 day old and three adult male Sprague-Dawley (SD) rats (180 g–220 g) were purchased from the Experimental Animal Center at Nantong University, China. The rats were specific pathogen free, originally from Charles River Laboratories (Wilmington, MA) and bred in Laboratory Animal Research Center at Nantong University. The animals were housed, in polycarbonate cages with corn cob beddings, in a 12-h light/dark schedule with ad libitum access to food and water in a barrier unit. All animal experiments were performed in accordance with the National Institutes of Health (NIH) Guide for the Care and Use of Laboratory Animals and approved by the Administration Committee of Experimental Animals of Nantong University, China (approval No. 20130410–006).

### SCs isolation and transfection

Rat SCs were harvested as previously described [43] with minor modifications. Briefly, the Sprague-Dawley rats (1 to 3 d- old) were sanitized using 75% ethanol prior to decapitation. Then sciatic nerves were harvested and enzymatically dissociated by incubation at 37 °C sequentially with 1% collagenase and 0.125% trypsin for 30 and 10 min, respectively. The mixture was triturated, centrifuged, and resuspended in 10% FBS in DMEM. The cell pellets were plated on poly-L-lysine precoated dishes using the same media. The following day, 10 μM cytosine arabinoside was added and incubated for an additional 48 h to remove fibroblasts. The cell culture was maintained in DMEM supplemented with 10% FBS, 2 μM forskolin (Sigma Aldrich, St. Louis, MO, USA), and 2 ng/ml heregulin (HRG, R&D system, Minneapolis, MN, USA) to stimulate SC proliferation. For additional purification, the cell culture was gently trypsinized, pelleted, and incubated with anti-Thy1.1 antibody (1:1000, Sigma Aldrich, St. Louis, MO, USA; Cat# M7898, RRID: AB_477242; Clone number: TN26) on ice for 2 h, followed by incubation in complement (Sigma Aldrich, St. Louis, MO, USA) for an additional 2 h. All media and supplements were purchased from Gibco-Invitrogen (Carlsbad, CA, USA).

For cell transfection, purified primary SCs were transfected with Cyr61 siRNAs designated as ((Cyr61-siRNA-1 (sequence: GCAGACCCTGTGAATATAA), Cyr61-siRNA-2 (sequence: GGAATGGGTCTGTGATGAA), Cyr61-siRNA-3 (sequence: GCTCCAGTGTGAAGAAATA)) or NTC (Ribobio, Guangzhou, Guangdong, China), using riboFECT CP Transfection kit (Ribobio, Guangzhou, Guangdong, China) following the manufacturer’s instructions.

### Nerve tissue preparation

The adult male SD rats were anesthetized intraperitoneally using a mixture of 85 mg/kg trichloroacetaldehyde monohydrate (RichJoint, Shanghai, China), 42 mg/kg magnesium sulfate (Xilong Scientific, Guangzhou, Guangdong, China), and 17 mg/kg sodium pentobarbital (Sigma Aldrich, St. Louis, MO, USA). After anaesthetization, the rats were transcranial perfused sequentially with saline and 4% (v/v) paraformaldehyde in 0.1 M PBS. Then, the sciatic nerve segments at 10 mm above the bifurcation into the tibial and common fibular nerves were harvested for frozen sections.

### Enzyme-Linked Immunosorbent Assay (ELISA)

Primary SCs were transfected with NTC or siRNA targeting Cyr61. After transfection, the media was replaced with serum-free medium for an additional 12 h incubation. The media was then harvested and filtered through a 0.22 μm filter (Millipore, Bedford, MA, USA). The protein levels of Cyr61 in the media were measured using a Cyr61 ELISA Kit (Cusbio, Wuhan, Hubei, China) based on the manufacturer’s instructions. Measurement data were summarized from 3 independent experiments, each run in triplicate.

### RNA extraction and quantitative real time RT-PCR (qPCR)

Total RNA of each group was extracted using Trizol (Invitrogen, Carlsbad, CA). Reverse transcription was carried out with SuperScript First-Strand Synthesis System (Invitrogen, Carlsbad, CA). Gene products were analyzed using Fast EvaGreen qPCR Master Mix (Biotium, Hayward, CA) and specific primers in StepOne Real-Time PCR System (Applied Biosystems). Reaction components in each well were composed of 2× Fast Eva Green Master Mix, 10 μl; primers, 1 μl each; template, 1 μl; ROX, 2 μl; and H_2_O, 5 μl. Three step fast cycling protocol was performed. Relative gene expression levels were calculated as ratios of the mRNA levels normalized against those of 18 s mRNA. All the results were expressed as the mean ± SD of three independent experiments. Primer sequences are provided in Additional file 1: Table 1.

### Western blot analysis

Total proteins from SCs were extracted using the M-PER cell protein extraction reagent (Pierce, Rockford, IL, USA). Extracted proteins were quantified using the Fast Silver Stain Kit (Beyotime, Haimen, Jiangsu Province, China). Twenty microgram of total protein were loaded onto a 12% (w/v) SDS-PAGE, electrophoresed, and transferred to a PVDF membrane (Millipore, Bedford, MA). After blocking for 1 h with 5% (w/v) non-fat dry milk in TBS-T (0.05% (v/v) Tween 20 in Tris-buffered saline), the membrane was incubated with specific primary antibodies diluted in blocking buffer overnight at 4 °C. The rabbit polyclonal antibody to Cyr61 (1:500, Abcam, Cambridge, MA, USA; Cat# ab24448, RRID: AB_2088724) and rabbit monoclonal antibody to c-Jun (1:2000, Abcam, Cambridge, MA, USA; Abcam Cat# ab40766, RRID: AB_731602, Clone number: EP693Y) were used. Afterward, the membranes were washed with TBS-T and then incubated with HRP conjugated secondary antibody diluted in blocking buffer (1:5000, Abcam, Cambridge, MA, USA) at RT for 2 h. Immunoreactive bands were visualized using enhanced chemiluminescence (Beyotime, Haimen, Jiangsu Province, China). Densitometry analysis was performed using the Image J software (http://imagej.nih.gov/ij/).

### Immunofluorescent staining

Cells were plated on poly-L-lysine pre-coated coverslips and cultured overnight. They were then fixed in 4% paraformaldehyde for 30 min at room temperature (RT). Sciatic nerve segments from adult rats were dissected, fixed in 4% paraformaldehyde for 24 h, dehydrated in 30% sucrose at 4 °C, then cut and mounted onto microscope slides. Cells and sciatic nerve sections were blocked for 2 h at 37 °C. Cells were incubated with the following antibodies overnight at 4 °C: mouse monoclonal or rabbit polyclonal antibody to S100β (1:100, Abcam, Cambridge, MA, USA; Cat# ab14849, RRID: AB_301508, Clone number: 4B3; Cat# ab52642, RRID: AB_882426), rabbit polyclonal antibody to Cyr61 (1:200, Abcam, Cambridge, MA, USA; Cat# ab24448, RRID: AB_2088724) or mouse monoclonal antibody to αvβ3 (1:200, R&D system, Minneapolis, MN, USA; Cat# MAB3050, RRID: AB_2128187; Clone number: #23C6). After washing, the cells and sciatic nerve sections were incubated with FITC-conjugated rabbit anti-mouse IgG and Cy3-conjugated donkey anti-rabbit IgG (1:400, Abcam, Cambridge, MA; Cat# ab6724, RRID: AB_955315; Cat# ab97075, RRID: AB_10679955) for 2 h at RT. Nuclei were counterstained with Hoechst 33342 dye (5 μg/mL, Sigma Aldrich, St. Louis, MO, USA). Fluorescence was visualized under a TCS SP5 confocal microscope (Leica Microsystems, Wetzlar, Germany).

### Cell proliferation analysis

SC proliferation was assessed after siRNA transfection, or exposure to recombinant Cyr61 with/without αvβ3 neutralizing antibody (R&D system, Minneapolis, MN, USA; Cat# MAB3050, RRID: AB_2128187; Clone number: #23C6) and mouse IgG1 isotype control (R & D, Minneapolis, MN, USA; Cat# MAB002, RRID: AB_357344; Clone number:11711) was used as a control for neutralizing antibody. Cell Counting Kit8 (CCK-8) (Biyuntian Company, Jiangsu Province, China) was then used. Briefly, an equal number of cells were plated onto a 96-well plate. Cells in each treatment group were cultured for 0-60 h. Then, 10 μL of CCK-8 solution was added to each well and incubated at 37 °C for an additional 2 h. Optical density (OD) was determined at a wavelength of 450 nm.

### Cell migration analysis

SC migration was monitored after siRNA transfection, or exposure to recombinant Cyr61 with/without neutralizing antibody and mouse IgG1 isotype control was used as a control for neutralizing antibody. The transwell migration assay was used as previously described [44]. After transfection, 2 × 10^4^ cells in serum-free DMEM were plated onto the upper chamber of each transwell with 8 μm pore size (Costar, Corning, Inc., NY). The lower chamber was supplemented with 800 μL of complete media (DMEM+ 10%FBS) or complete media with Cyr61 or complete media with Cyr61 and αvβ3 neutralizing antibodies. Cells were incubated for 24 h at 37 °C in 5% CO_2_. Non-migrating cells were removed from the upper surface of the membrane using a cotton swab. Cells on the lower side of the membrane were stained with crystal violet, and migration was quantified by counting cells from four microscope fields. Each treatment condition was run in triplicate.

### Statistical analysis

Data were expressed as mean ± SEM, and statistical analysis was performed using GraphPad Prism Software (GraphPad Software, LaJolla, CA). Comparisons between two groups were performed using the Student’s t-test. Differences of *p* < 0.05 were considered statistically significant.

## Supplementary Information

**Additional file 1: Table 1.** Primers used for qPCR analysis.

**Additional file 2: Figure S1.** Uncropped images of Western blots used in figures.

## Data Availability

The complete blots of relevant figures are provided in Additional file 2. Further datasets used and/or analysed during the current study available from the corresponding author on reasonable request.

## Abbreviations

Cyr61/CCN1: Cysteine-rich 61
DMEM: Dulbecco’s Modified Eagle Medium
ELISA: Enzyme-linked immunosorbent assay
FBS: Fetal bovine serum
HRG: Heregulin
ICF: Immunocytofluorescence
IHF: Immunohistofluorescence
NTC: Non-targeting negative control
OD: Optical density
PNS: Peripheral nerve system
qPCR: Quantitative Real Time RT-PCR
RT: Room temperature
SCs: Schwann cells
SD: Sprague-Dawley
