# Supplementary figures and images for: Cyr61 promotes Schwann cell proliferation and migration via αvβ3 integrin
Source: BMC Mol Cell Biol. 2021 Apr 7;22:21. doi: 10.1186/s12860-021-00360-y (PMC8028786; doi:10.1186/s12860-021-00360-y)

Figure1C

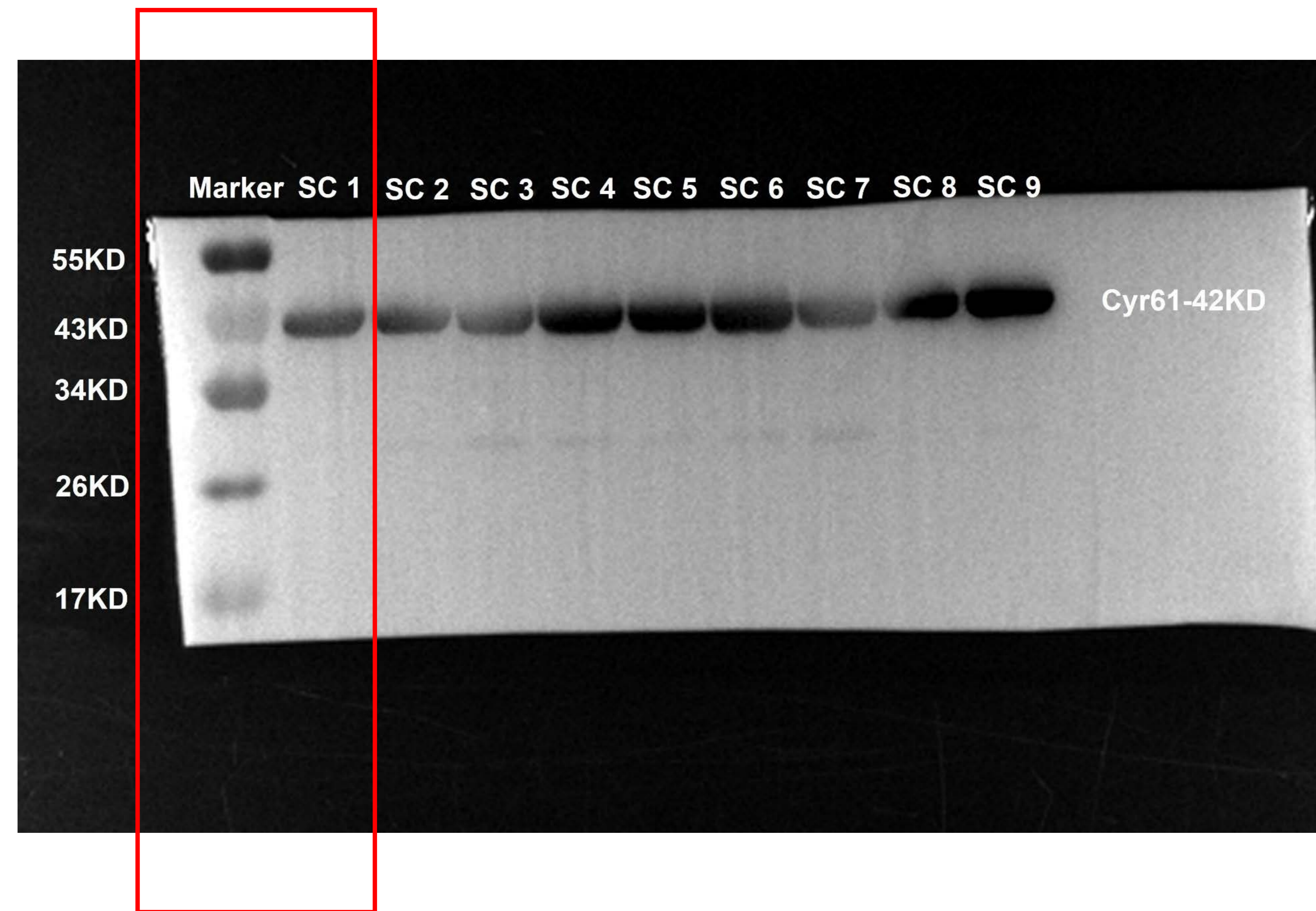

Figure2B

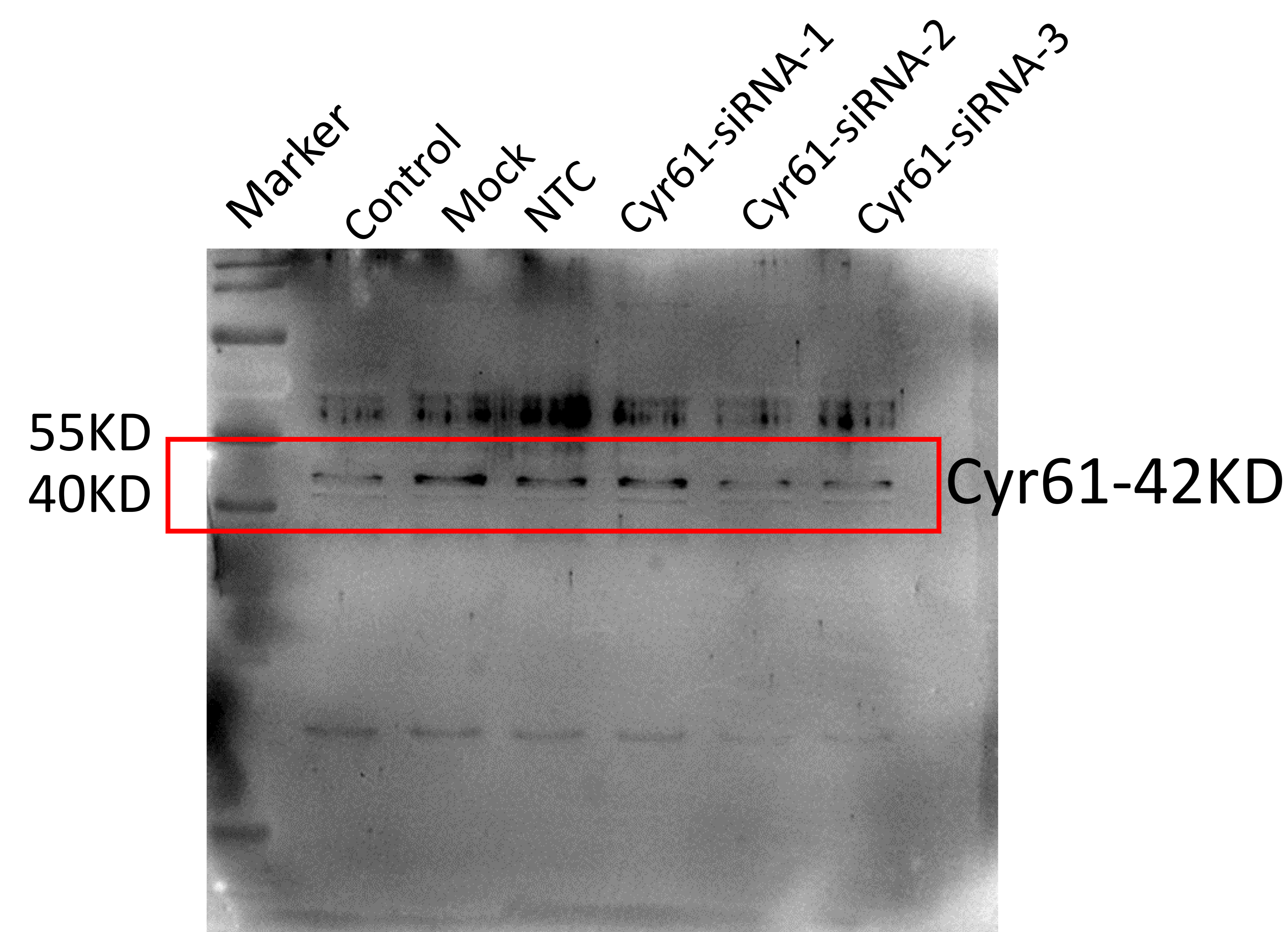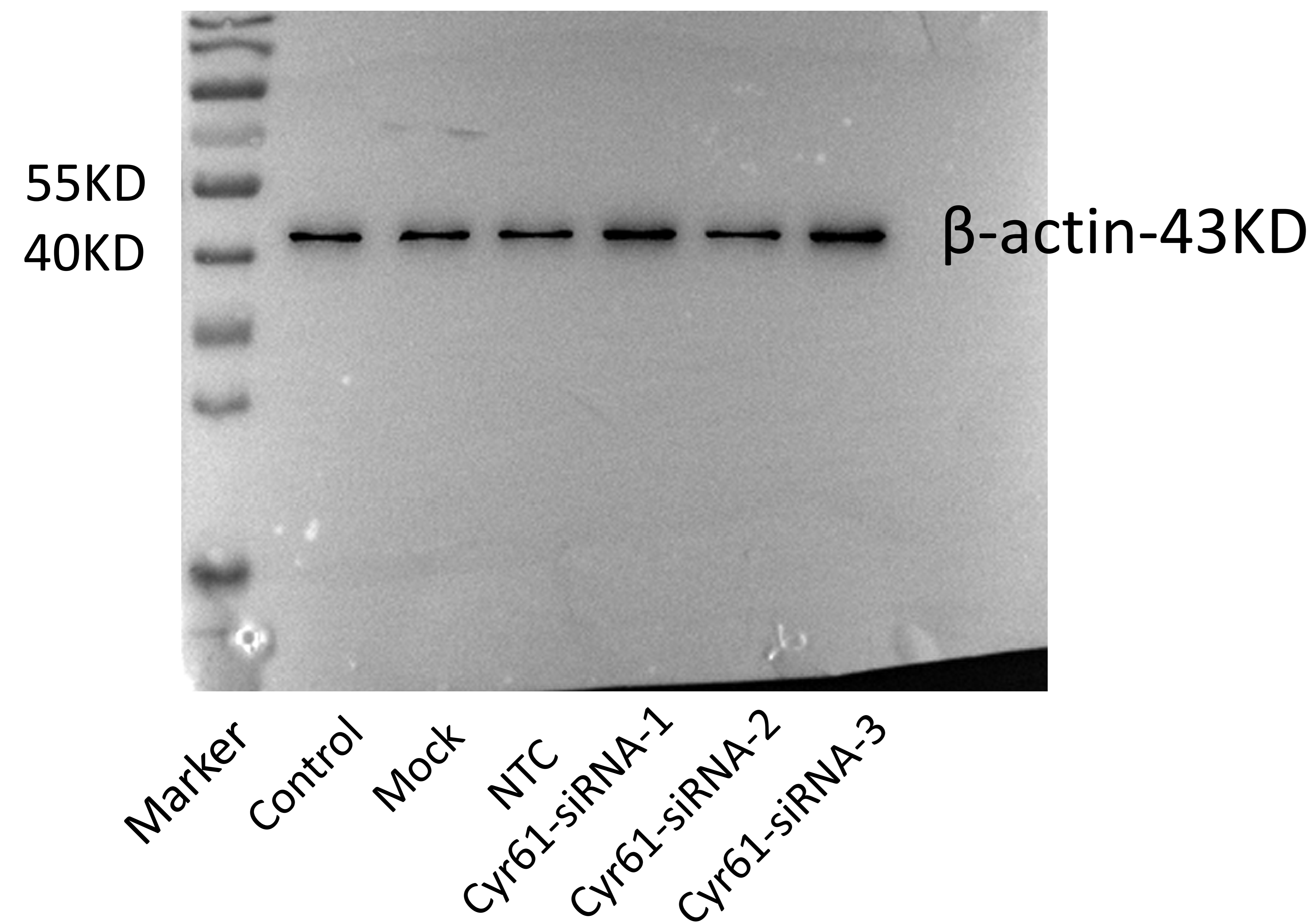

Figure2H

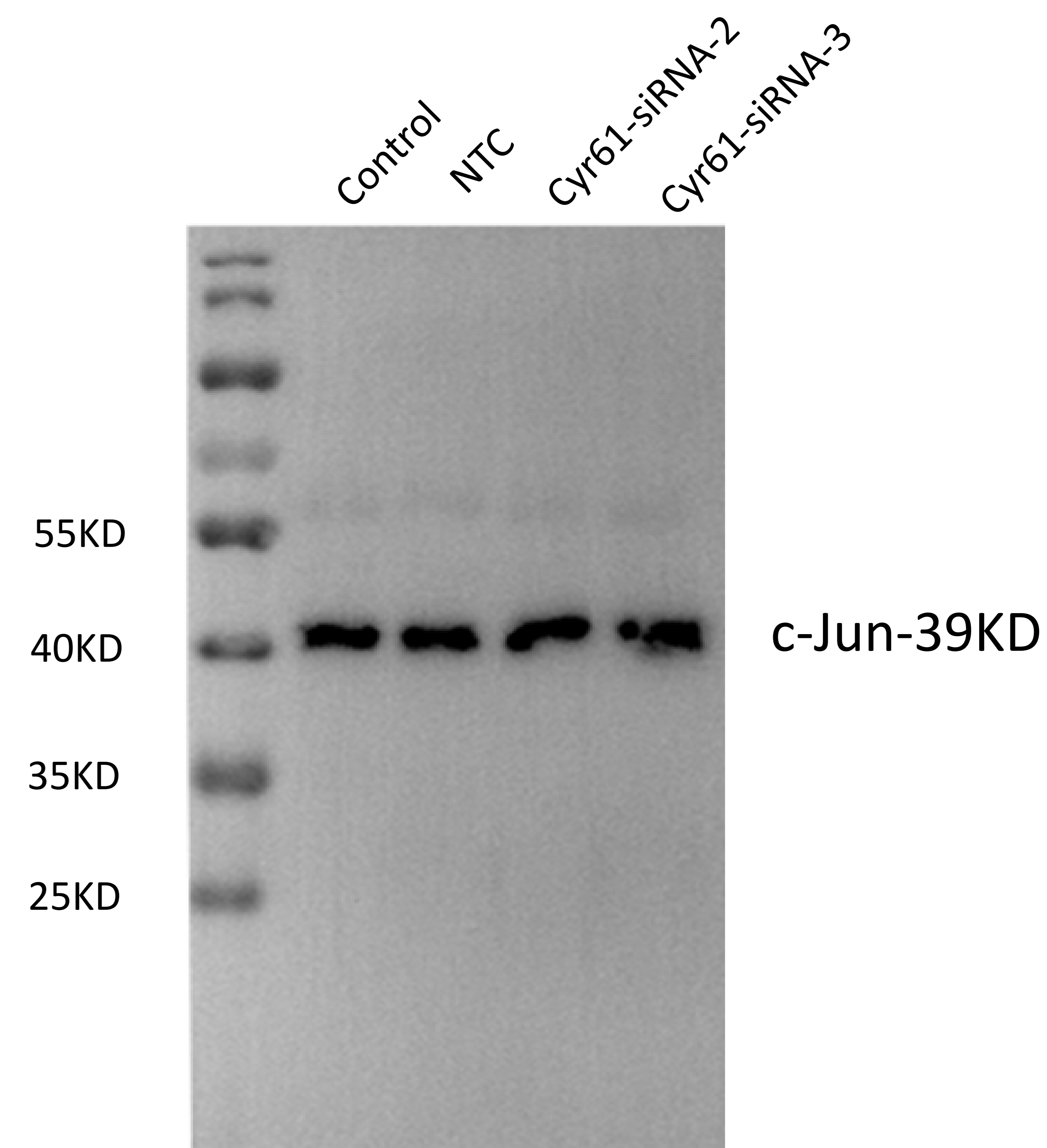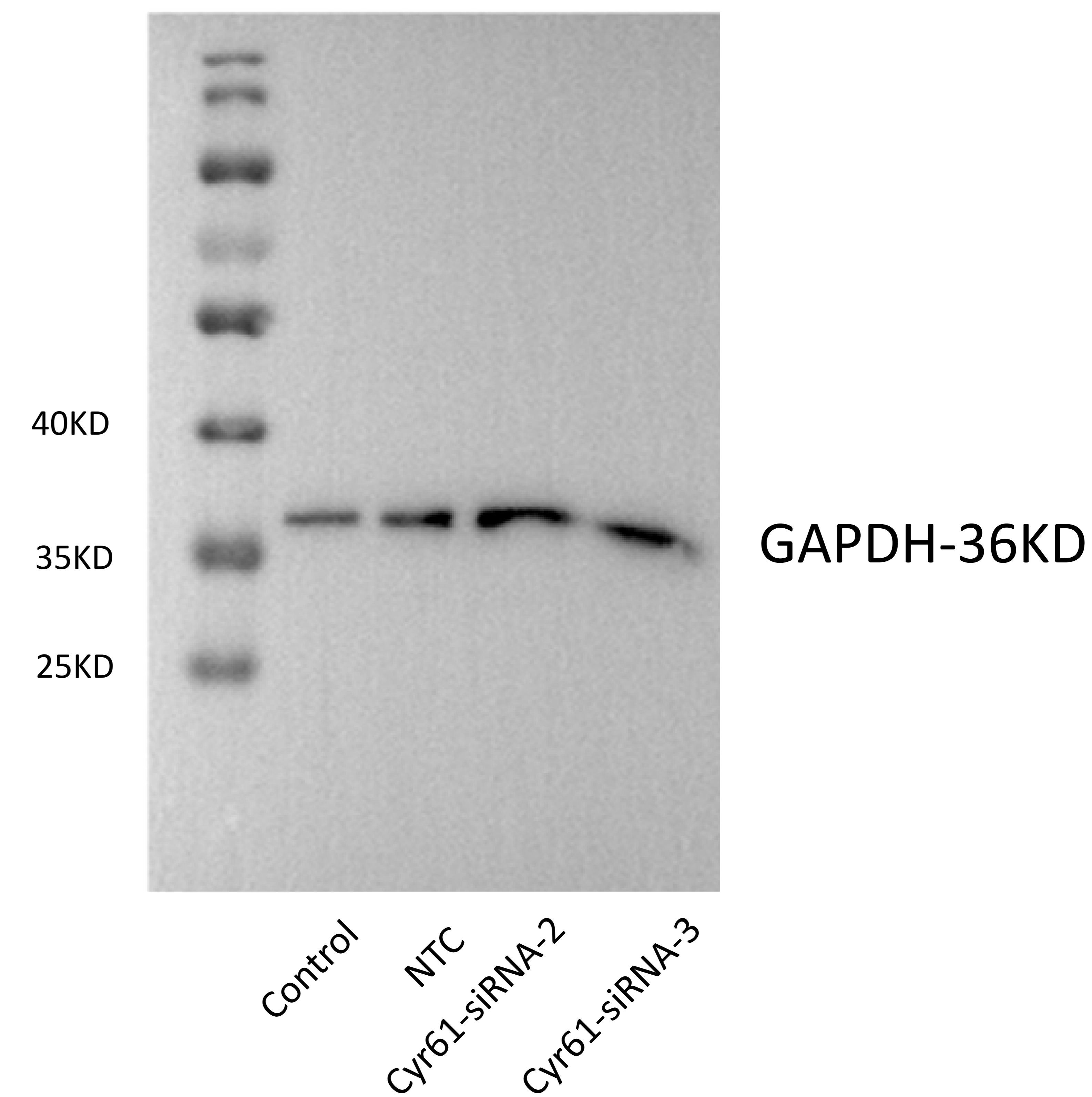

Figure3F

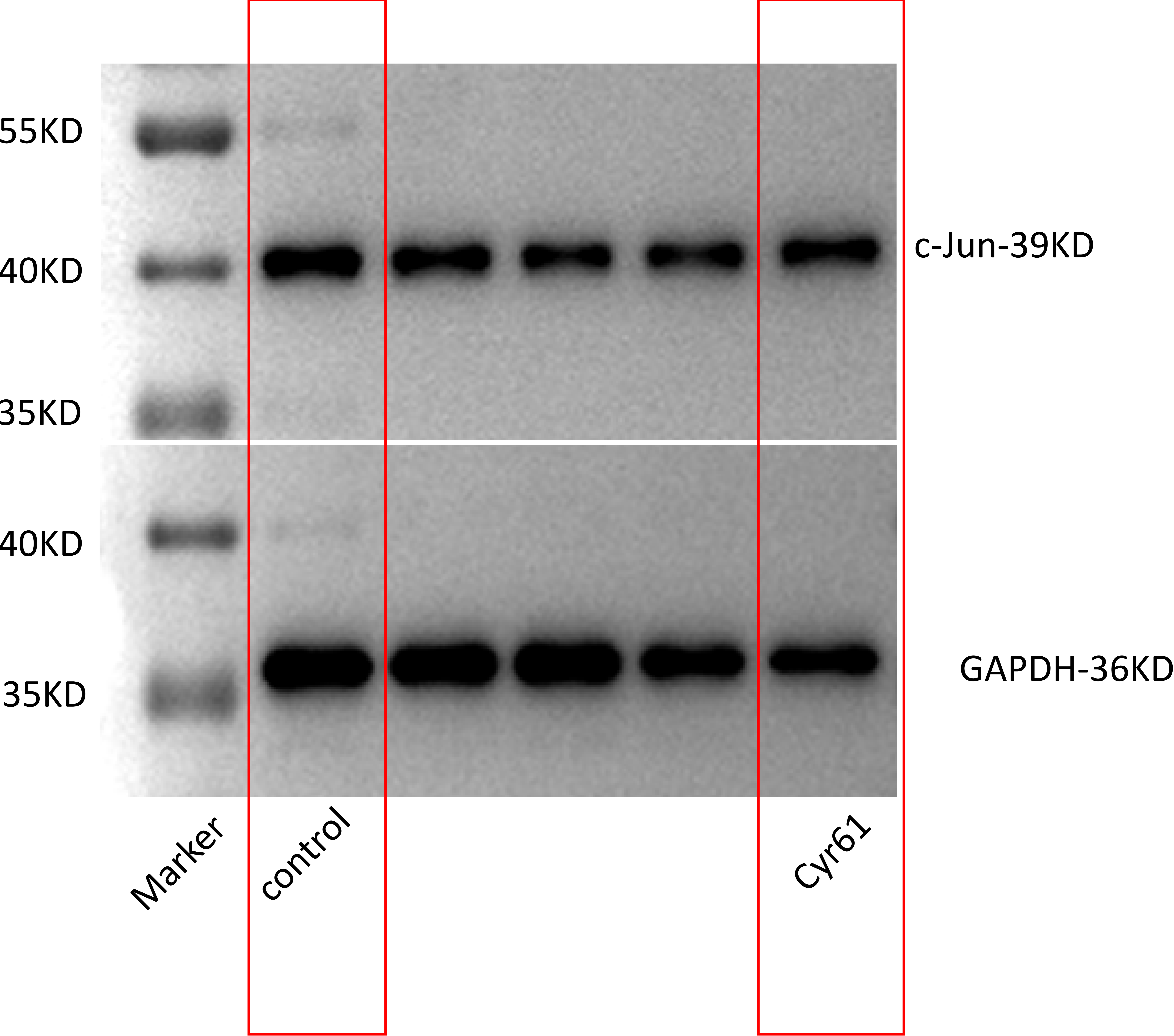

Supplement: Supplementary file 2 — Additional file 2: Figure S1. Uncropped images of Western blots used in figures. [file 12860_2021_360_MOESM2_ESM.pdf]
